# Effect of Glycerol-Induced Hyperhydration on a 5-kilometer Running Time-Trial Performance in the Heat in Recreationally Active Individuals

**DOI:** 10.3390/nu15030599

**Published:** 2023-01-24

**Authors:** Antoine Jolicoeur Desroches, Catherine Naulleau, Thomas A. Deshayes, Hugo Parent-Roberge, Timothée Pancrate, Eric D. B. Goulet

**Affiliations:** 1 Faculty of Physical Activity Sciences, University of Sherbrooke, Sherbrooke, QC J1K 2R1, Canada; 2 Institut National du Sport du Québec, Montréal, QC H1V 3N7, Canada; 3 Research Centre on Aging, University of Sherbrooke, Sherbrooke, QC J1H 4C4, Canada

**Keywords:** hyperhydration, hydration, glycerol, performance, running

## Abstract

Maximal oxygen consumption ($\dot{V}$O_2max_) is a major determinant of 5-km running time-trial (TT) performance. Glycerol-induced hyperhydration (GIH) could improve $\dot{V}$O_2max_ in recreationally active persons through an optimal increase in plasma volume. Moreover, ingestion of a large bolus of cold fluid before exercise could decrease thermal stress during exercise, potentially contributing to improved performance. We determined the effect of GIH on 5-km running TT performance in 10 recreationally active individuals (age: 24 ± 4 years; $\dot{V}$O_2max_: 48 ± 3 mL/kg/min). Using a randomized and counterbalanced protocol, participants underwent two, 120-min hydration protocols where they ingested a 1) 30 mL/kg fat-free mass (FFM) of cold water (~4 °C) with an artificial sweetener + 1.4 g glycerol/kg FFM over the first 60 min (GIH) or 2) 7.5 mL/kg FFM of cold water with an artificial sweetener over the first 20 min (EUH). Following GIH and EUH, participants underwent a 5-km running TT at 30 °C and 50% relative humidity. After 120 min, GIH was associated with significantly greater fluid retention (846 ± 415 mL) and plasma volume changes (10.1 ± 8.4%) than EUH, but gastrointestinal (GI) temperature did not differ. During exercise, 5-km running TT performance (GIH: 22.95 ± 2.62; EUH: 22.52 ± 2.74 min), as well as heart rate, GI temperature and perceived exertion did not significantly differ between conditions. This study demonstrates that the additional body water and plasma volume gains provided by GIH do not improve 5-km running TT performance in the heat in recreationally active individuals.

## 1. Introduction

Maximal oxygen consumption ($\dot{V}$O_2max_) is a determinant factor of 5-km running time-trial (TT) performance. Indeed, Ramsbottom et al. [1] demonstrated that a 5-km running TT performance can be explained, at least in large part, by the magnitude of $\dot{V}$O_2max_. This observation is unsurprising, as an all-out 5-km running TT is completed at approximately 90% of $\dot{V}$O_2max_ and 98% of maximum heart rate [2]. The Fick equation dictates that the $\dot{V}$O_2max_ depends on both the cardiac output and the arteriovenous oxygen difference [3]. Therefore, it is reasonable to believe that any positive alteration in $\dot{V}$O_2max_, either by manipulating cardiac output, or arteriovenous oxygen difference, or both, could potentially lead to an improved 5-km running TT performance. It is possible to increase cardiac output by artificially increasing plasma volume [4]. A simple and legal way to enhance plasma volume is to induce hyperhydration prior to exercise using glycerol. Indeed, glycerol-induced hyperhydration (GIH) has been shown to increase total body water by ~ 800 mL and plasma volume by ~ 8% [5,6,7] 2 h following the ingestion of 26 mL of water/kg body mass (BM) with 1.2 g of glycerol/kg BM.

The capacity of an increase in plasma volume to enhance $\dot{V}$O_2max_ appears to be dependent upon an individual’s level of training and the magnitude of the increase in plasma volume [8]. Indeed, in general, this blood manipulation has been shown to be advantageous (1) in sedentary or recreationally trained individuals [9] and (2) when an increase in plasma volume of the order of 7–8% or 200–300 mL is generated [10]. The plasma volume of healthy people weighing 60–75 kg with an average hematocrit of 40% is estimated to be approximately 2520–3150 mL [11]. Therefore, an increase in plasma volume of 8% in this population would generate a theoretical increase in plasma volume of the order of 200 to 250 mL. Thus, it is reasonable to believe that the use of pre-exercise GIH could lead to an increase in $\dot{V}$O_2max_. In this regard, Patlar et al. [12] demonstrated that GIH improves $\dot{V}$O_2max_ both in sedentary and exercising individuals, likely through an optimal augmentation of cardiac output as there is no reason to believe that, mechanistically, GIH could widen arteriovenous oxygen difference.

In addition, decreased hyperthermia during exercise may help endurance performance [13]. To this effect, at any given exercise intensity during exercise conducted under warm ambient conditions, an increased plasma volume may lead to a more favorable distribution of blood to the working muscles and skin, thereby favoring heat dissipation and improved performance [14]. On the other hand, the ingestion of 26 mL of water/kg BM provided at 4 °C would be expected to result in a drop in core body temperature, compared to a pre-exercise euhydration state [15,16].

All observations provided above lead us to believe that GIH may be advantageous for endurance performance under warm ambient conditions. Therefore, the purpose of this study was to compare the effect of pre-exercise GIH to a state of pre-exercise euhydration (EUH) on fluid balance responses, performance, gastrointestinal (GI) temperature, heart rate and rating of perceived exertion during a 5-km running TT conducted in a warm environment in recreationally active individuals. We hypothesized that (1) GIH would produce a state of hyperhydration of at least 800 mL; (2) the increase in plasma volume at the beginning of exercise would be more important and its decline less important immediately following exercise with GIH than EUH; (3) there would be no significant difference in heart rate and rating of perceived exertion during the 5-km running TT between conditions; (4) the GI temperature would be lower with GIH than EUH throughout the hydration and exercise periods; and (5) the 5-km running TT time would be faster with GIH than EUH.

## 2. Materials and Methods

### 2.1. Participants

Ten (9 men; 1 woman) recreationally active individuals (mean ± SD: 24 ± 4 years; 175 ± 10 cm; 69.5 ± 9.3 kg; 13.6 ± 6.7% body fat; 60.3 ± 9.4 kg fat-free-mass (FFM); 190 ± 9 bpm for maximal heart rate; 48 ± 3 mL/kg/min for $\dot{V}$O_2max_) participated in this study. Inclusion criteria were: (1) being healthy and between the age of 18–50 years, (2) training at least 3 h per week, (3) practicing an aerobic sport for at least one year, (4) taking no medication that can affect core body temperature and hydration state and (5) having a body mass index < 30 kg/m^2^. The protocol was thoroughly explained, and participants gave their written informed consent to participate in this study. The CIUSSS Estrie-CHUS Ethics Committee (2020-3606) approved all experimental procedures.

### 2.2. Overview of the Study

The study used a randomized and counterbalanced protocol. Participants first underwent a preliminary visit where baseline measurements were taken. No more than 10 days following the first visit, participants realized a familiarization trial to customize themselves with the 5-km running TT. Participants then underwent two experimental trials (GIH and EUH) separated by 7 days and conducted at the same time of day.

### 2.3. Preliminary Visit

During the preliminary visit, the height of the participants, wearing only socks, was measured to the nearest 0.5 cm using a wall stadiometer, post-void nude BM with a digital scale (BX-300+, Altron Systems, Mount Pleasant, SC, USA, ±20 g), fat mass and FFM with the dual-energy X-ray absorptiometry technology (Lunar Prodigy, GE Healthcare, Chicago, IL, USA) and blood pressure and resting heart rate using a digital sphygmomanometer (Welch-Allyn 420 series, Skaneateles Falls, NY, USA) after the participants remained seated for a period of 2 min. Finally, $\dot{V}$O_2max_ was determined with a metabolic analyzer (Cosmed Quark CPET, Cosmed, Chicago, IL, USA) calibrated according to the manufacturer instructions using an incremental running protocol on a motorized treadmill. Participants started walking at 5 km/h with 0% grade for 1 min, with further increments of 1 km/h occurring every 1 min until the participants could not continue. The $\dot{V}$O_2max_ was confirmed when at least two of those criteria were reached: (1) respiratory exchange ratio ≥ 1.1, (2) theoretical maximal heart rate (220-age), (3) $\dot{V}$O_2_ plateau concurrent to an increase in running speed [17].

### 2.4. Pre-Experimental Protocol

For the 24-h period preceding the familiarization trial, participants filled a dietary log (nutrition and hydration), which they replicated for the 24-h period preceding the two experimentations. Participants refrained from consuming diuretics, with the exception of caffeine, during the 24-h period preceding the experimentations as well as from taking any supplements in the 48-h period preceding the experimentations [5,6,7]. To optimize hydration before the experimentations and familiarization trial, participants drank 250 mL of water 120 min before going to sleep the night before these visits and 250 mL 60 min before arriving at the laboratory [5,6,7]. Both before the experiments and the familiarization trial, participants refrained from eating and drinking in the 60-min period preceding their arrival at the laboratory, went to bed at the same time of day the preceding nights and maintained their training routine over the last 24-h period, while stopping any form of exercise for the last 8 h leading to these visits [7].

### 2.5. Familiarization Trial

A familiarization trial was executed to minimize any learning effect [18]. During this visit, participants underwent the 5-km running TT under the same experimental conditions as those used during the experiments, with the exception that they were not required to ingest the GI pill or to undergo the hydration protocols beforehand.

### 2.6. Experimental Trials

**Figure 1:**
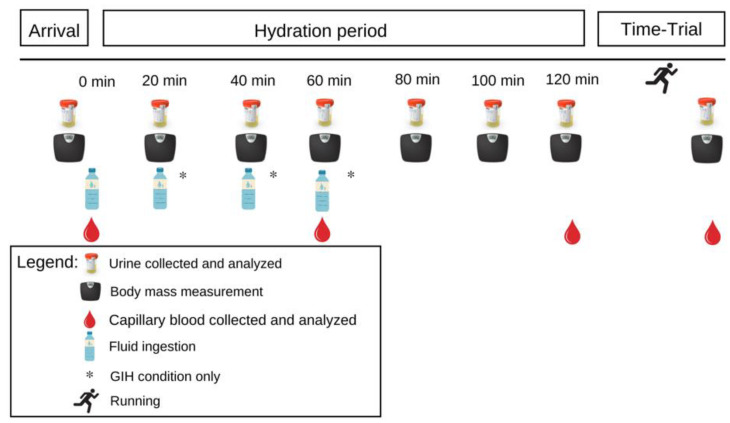
Illustration of the experimental protocol.

Each experimental trial was divided into three phases: (1) arrival at the laboratory and baseline data collection, (2) 120-min hydration period and, (3) 5-km running TT. Figure 1 illustrates all phases of the experimental protocol. At their arrival at the laboratory, participants voided their bladder in a graded urinal. Participants were then weighed and instrumented with a heart rate monitor after which they were seated for 10 min with their hand immersed in 40 °C water. A capillary blood punction was then performed and measurements of heart rate, GI temperature and subjective perceptions were taken.

Then, the participants started the 120-min hydration period, which consisted of ingesting either (1) 7.5 mL/kg FFM of cold water (~4 °C) with an artificial sweetener + 1.4 g glycerol/kg FFM at times 0, 20, 40 and 60 (GIH) [5,6,7] or (2) 7.5 mL/kg FFM of cold water with an artificial sweetener at time 0 only (EUH). Every 20 min up until min 120, heart rate, GI temperature and subjective perceptions were measured and then participants urinated, urine was collected and BM was measured. With the exception of when they urinated and were weighed, participants always remained seated. Capillary blood samples were collected at minutes 60 and 120; they occurred prior to standing to limit the impact of body posture on plasma volume changes and following 10 min of hand immersion in 40 °C water. Skin thermistors were installed on participants over the last 20-min period of the hydration period.

Following the hydration period, participants were transferred to the environmental chamber where they mount the motorized treadmill and remained silent for 2 min. Then, the participants ran at a pace of their choice on the treadmill for a 5-min period to warm up before the TT. The participants’ heart rates, GI and skin temperatures and subjective perceptions were measured before and after the warmup and then the 5-km running TT started at 0% grade, after the participants had recovered for 2 min. The participants could adjust their speed throughout the running period and did not have access to any metrics, such as speed and elapsed time, except for the distance completed. Standardized verbal encouragement was provided during the TT. Two propeller-type fans (24″ diameter, 8800 CFM, Secco international, St-Hyacinthe, QC, Canada) as well as an axial-type fan (13″ diameter, 1720 CFM, Maximum Canada, Toronto, ON, Canada) simulated wind in front of participants at a velocity closely approximating running speed. Wind speed was verified using an anemometer (DAF800, General Tools & Instruments, Secaucus, NJ, USA). Heart rate, GI, skin temperatures and subjective perceptions were measured at the end of each km. A capillary blood sample was taken immediately following the TT. Fluid was not provided during exercise.

### 2.7. Measurements

#### 2.7.1. Heart Rate and Gastrointestinal and Skin Temperatures

Heart rate was measured with a Garmin Premium heart rate monitor (Garmin, Olathe, KS, USA). Gastrointestinal temperature was measured using calibrated telemetric pills (CoreTemp, Palmetto, FL, USA) ingested 10 h prior to the participants’ arrival at the laboratory [19]. Skin temperature was measured with calibrated YSI 409 B probes (Yellow Springs Instrument, Yellow Springs, OH, USA) placed on the left side of the body on the chest, the forearm, the thigh and the calf. Hypafix dressing tape was used to hold the probes in place. The skin probes were connected to a USB-TEMP data acquisition box (MC measurement computing, Norton, MA, USA). Mean skin temperature was calculated according to Ramanathan [20] using the following Equation (1):

T_skin_ = (0.3 × T_chest_) + (0.3 × T_forearm_) + (0.2 × T_thigh_) + (0.2 × T_calf_), (1)

where T is temperature.

#### 2.7.2. Urinary Measurements, Assessment of Accumulated Fluid Retention and Excretion, and Sweat Loss

Urine volume was measured gravimetrically using a digital scale (Symmetry, Cole-Parmer, QC, Canada) considering that 1 mL of urine equals 1 g. Urine specific gravity was measured using a refractometer (PAL-10S, Atago, Bellevue, WA, USA), and urine osmolality was measured using the freezing point depression technique (Micro-Osmometer, Osmette, Precision Systems Inc., Natick, MA, USA). Accumulated fluid retention observed every 20 min during the hydration period was calculated using the following Equation (2) [5,6,7]:

Accumulated fluid retention from the previous timepoint (mL) + fluid consumed during the previous timepoint (mL) − urine produced at the given timepoint (mL)(2)

Accumulated urine volume represented the sum of each volume of urine produced during each timepoint. Sweat loss during the TT was determined by subtracting the post- from the pre-TT BM [18]. Losses of mass associated with the respiratory exchange of O_2_ and CO_2_, as well as respiratory water losses were not considered in the calculation of the sweat loss during the TT and were assumed to be similar among the two experimentations [21]. Sweat loss was corrected by exercise time (h) to obtain sweat rate.

#### 2.7.3. Capillary Blood Measurements

This procedure was executed as reported in Goulet et al. [5]. The finger was first cleaned and disinfected with 70% isopropyl alcohol. Then, a high blood flow lancing device (Capiject, Terumo, Vaughan, ON, Canada) was used to prick the finger at a depth of ~2 mm. After the first blood drop had been swiped away, ~400 μL of blood was collected in a capillary lithium-heparin tube (BD microtainer, Mississauga, ON, Canada). Blood was collected to measure hemoglobin levels using the Alere H2 Hemopoint system (Alere, Lowell, MA, USA), hematocrit—the centrifugation technique, osmolality—the freezing point depression technique (Micro-Osmometer, Osmette, Precision Systems Inc., Natick, MA, USA) and natremia—the ion selective electrode technique (Medica EasyElectrolytes, Fisher Scientific, Pittsburg, PA, USA). Capillary hemoglobin concentration and hematocrit values have been shown to be highly correlated to venous blood hemoglobin and hematocrit values [22]. The Dill and Costill [23] equation was used to estimate plasma volume changes. Measurements were performed in duplicate.

#### 2.7.4. Subjective Perceptions

Perceived thirst (1–11 scale) [24] and heat stress (1–7 scale) [25] were both measured during the hydration and exercise periods, perceived abdominal pain and bloating only during the hydration period (1–5 scale) [6] and perceived exertion (6–20 scale) [26] only during the exercise period.

### 2.8. Statistical Analysis

All statistical analyses were performed using the IBM SPSS Statistics software (version 28, New York, NY, USA). Shapiro–Wilk tests were used to analyze data normality. Dependent t-tests were used to compare results pertaining to hydration state at arrival at the laboratory (BM, urine osmolality, blood natremia and osmolality) and environmental conditions (relative humidity, temperature and simulated wind speed). When normality was not respected, which was the case for urine specific gravity on arrival at the laboratory, a Wilcoxon test was executed to compare the two conditions. Effect sizes were computed for hemoglobin, plasma volume, heart rate, GI temperature, perceived exertion and the 5-km TT performance with this Equation (3) [27]:

(Mean time GIH (min) − mean time EUH (min))/standard deviation of EUH (3)

Two-factor (condition and time) repeated-measures ANOVAs were used to determine the time, condition and condition × time effects on variables repeatedly measured during the experiments. When sphericity was violated, Greenhouse–Geisser correction was applied. Multiple pairwise comparisons were executed when significant interaction effects were observed. Post-hoc corrections were performed using the false discovery rate procedure. To examine the impact of baseline hydration status on TT performance, a Pearson product-moment correlation was performed between the changes in USG between conditions at baseline and the changes in the 5-km TT performance. A Pearson product-moment correlation was also performed between $\dot{V}$O_2max_ and EUH-related 5-km TT performance. Statistical significance was set at *p* ≤ 0.05 and results are reported as means ± SD. A power computation indicated that based on an intra-subject test-retest standard deviation of 30 s for the 5-km running TT [28], a significance level of α = 0.05 and a minimal difference between the two conditions set at 30 s, an *n* = 10 was required to have an 80% chance to detect a statistically significant difference between the two conditions. Hence, 10 participants were recruited.

## 3. Results

### 3.1. State of Hydration of Participants at Arrival at the Laboratory and Ambient Conditions during the Hydration and 5-kilometer Running Time-Trial Periods

Table 1 shows the hydration state-associated variables. Based on BM, urine specific gravity and osmolality and blood osmolality and natremia values, participants were in a well and similarly hydrated state prior to both experimentations [29,30,31,32]. There was no significant difference in ambient temperature (GIH: 21.8 ± 0.3; EUH: 21.6 ± 0.2 °C; *p* = 0.089) and relative humidity (GIH: 58.8 ± 7.5; EUH: 59.6 ± 7.5%; *p* = 0.88) between conditions during the hydration periods. Temperature (GIH: 29.9 ± 0.2; EUH: 29.9 ± 0.1 °C; *p* = 0.86), relative humidity (GIH: 52.3 ± 3.3; EUH: 51.6 ± 1.5%; *p* = 0.27), and simulated wind speed (GIH: 14.09 ± 1.0; EUH: 15.0 ± 1.0 km/h; *p* = 0.38) during the TT inside the environmental chamber did not significantly differ between conditions.

### 3.2. Fluid Retention, Body Mass, Plasma Volume, Hemoglobin Concentration and Sweat Loss

**Figure 2:**
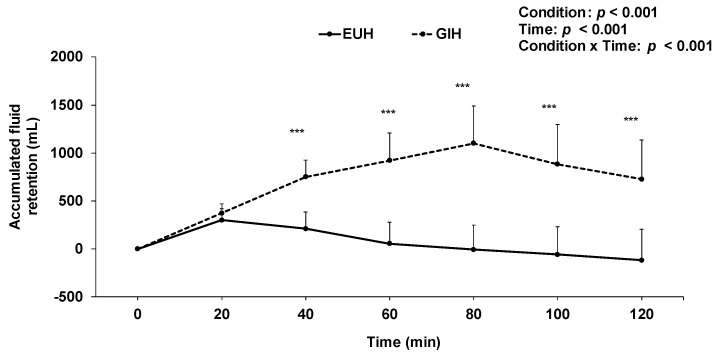
Effect of EUH and GIH on accumulated fluid retention during the hydration periods. *** *p* < 0.001 compared with EUH. EUH = euhydrated; GIH = glycerol-induced hyperhydration.

**Figure 3:**
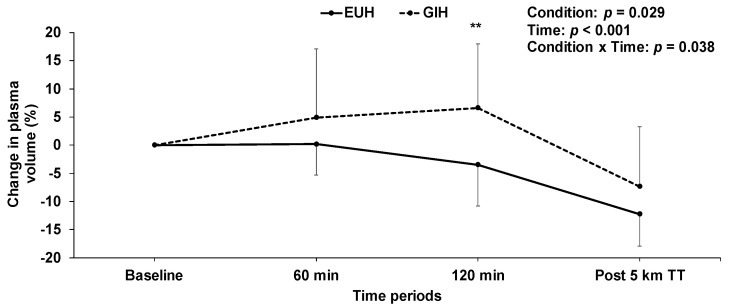
Effect of EUH and GIH on the changes in plasma volume over time during the hydration periods and time-trials. ** *p* < 0.01 compared with EUH. EUH = euhydrated; GIH = glycerol-induced hyperhydration; TT = time-trial.

At the end of the hydration period, accumulated fluid retention with GIH was 727 ± 408 mL, compared to −119 ± 325 mL (*p* < 0.001) with EUH (Figure 2). Consequently, as per study design, GIH was associated with 846 ± 415 mL more fluid retention than EUH at minute 120. Body mass was higher (*p* = 0.001) with GIH (69.7 ± 8.4 kg) than with EUH (68.6 ± 8.7 kg) at minute 120. Figure 3 shows the changes in plasma volume that occurred throughout the experimentations with GIH and EUH. Following the hydration period at minute 120, there was an increase in plasma volume of 6.6 ± 11.4% with GIH, whereas a decrease of 3.5 ± 7.3% was observed with EUH (*p* = 0.004). Hence, plasma volume was 10.1 ± 8.4% higher with GIH than with EUH after the hydration period (*p* = 0.004, effect size = 1.39). This increase in plasma volume resulted in a significant reduction in hemoglobin concentration (GIH: 14.7 ± 1.0; EUH: 15.6 ± 0.9 g/dL; *p* = 0.0002, effect size = −0.93) and hematocrit (GIH: 43.4 ± 3.2; EUH: 45.4 ± 3.0%; *p* = 0.007) in the GIH compared to the EUH condition. Although it was of a lesser magnitude with GIH, at the end of the TT, no significant difference in the decline of plasma volume between the two conditions was observed, compared with baseline (GIH: −7.3 ± 10.6; EUH: −12.3 ± 5.7%; *p* = 0.15, effect size = 0.88). At this timepoint, hemoglobin concentration was not significantly different between conditions (GIH: 15.8 ± 1.4; EUH: 16.2 ± 1.2 g/dL; *p* = 0.085, effect size = −0.33). The mean change in plasma volume from baseline during exercise was −7.9 ± 5.1% with EUH compared to −0.4 ± 10.2% with GIH (*p* = 0.02, effect size = 1.47). On the other hand, mean hemoglobin concentration values during exercise were 15.9 ± 1.0 with EUH compared to 15.2 ± 1.1 g/dL for GIH (*p* < 0.01, effect size = −0.7). Sweat loss (GIH: 586 ± 296; EUH: 590 ± 284 mL, *p* = 0.904) and sweat rate (GIH: 1.5 ± 0.8; EUH: 1.5 ± 0.6 L/h; *p* = 0.757) did not significantly differ between conditions during the TT.

### 3.3. Gastrointestinal and Mean Skin Temperatures and Heart Rate

**Figure 4:**
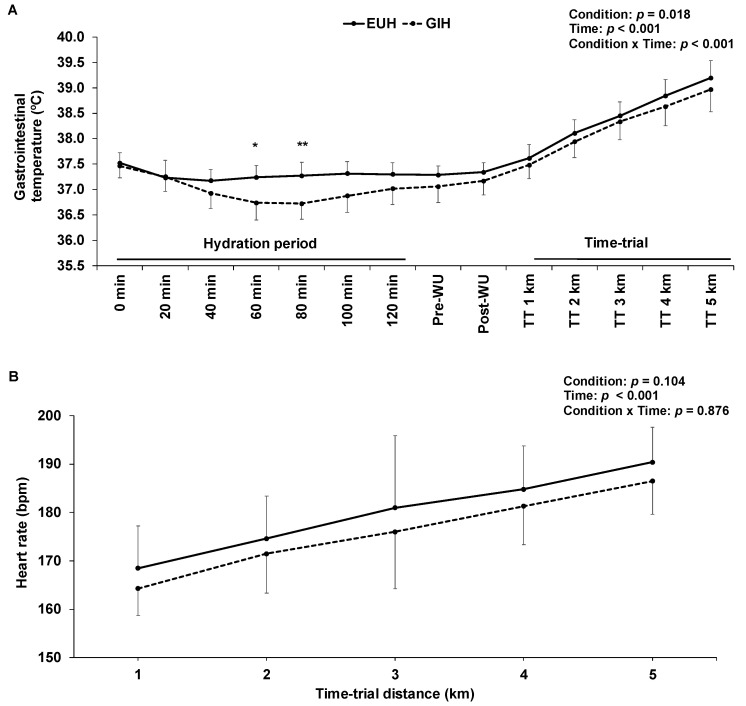
Effect of EUH and GIH on gastrointestinal temperature (**A**) over time during the hydration periods and time-trials and heart rate (**B**) during the time-trials only. * *p* < 0.05; ** *p* < 0.01 compared with EUH. EUH = euhydrated; GIH = glycerol-induced hyperhydration; TT = time-trial; WU = warmup.

The changes in GI temperature throughout time with GIH and EUH are illustrated in Figure 4A. A condition effect (*p* = 0.018) was observed, indicating that, on average, GI temperature was lower with GIH than with EUH. However, post-hoc analyses revealed that only at times 60 (*p* = 0.014) and 80 min (*p* = 0.007) of the hydration periods were GI temperatures lower with GIH than EUH. Therefore, there was no significant difference in GI temperature at the end of the hydration period between the two conditions (GIH: 37.0 ± 0.3; EUH: 37.3 ± 0.2 °C; *p* = 0.082, effect size = −1.5) nor at the end of the TT (GIH: 39.0 ± 0.4; EUH: 39.2 ± 0.3 °C; *p* = 0.12, effect size = −0.66). There were interaction (*p* = 0.026) and condition (*p* = 0.045) but not time effects (*p* = 0.145) for skin temperature during the TT. However, post-hoc analyses revealed that at no time during the TT were the differences between conditions statistically significant (*p* > 0.095). Heart rate continuously increased and similarly across time between conditions, with no interaction effect (*p* = 0.876), as illustrated in Figure 4B. The 5-km running TT was performed at 180 ± 11 and 176 ± 10 bpm (*p* = 0.10, effect size = −0.36), 94 ± 4 and 93 ± 3% of maximal heart rate (*p* = 0.10, effect size = −0.25) and 93 ± 6 and 90 ± 5% of $\dot{V}$O_2max_ (*p* = 0.11) with EUH and GIH, respectively.

### 3.4. Subjective Perceptions

**Figure 5:**
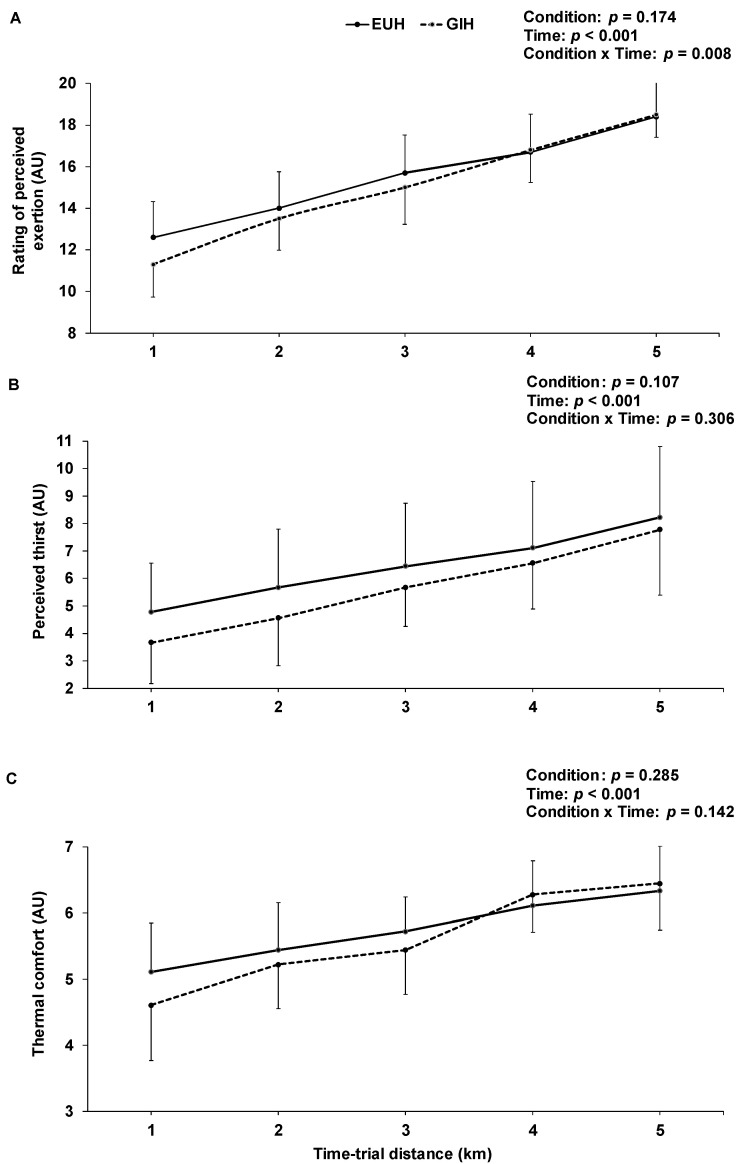
Effects of EUH and GIH on the rating of perceived exertion (**A**), perceived thirst (**B**) and thermal comfort (**C**) during the time-trials. AU = arbitrary units; EUH = euhydrated; GIH = glycerol-induced hyperhydration.

Rating of thirst was similar for GIH and EUH during the first hour of the hydration period, but became significantly lower with GIH during the second hour (all *p* < 0.03). Furthermore, rating of thermal comfort was significantly lower with GIH, compared to EUH, between the time 60 and 100 min of the hydration period (all *p* < 0.04) but was similar at the end of the hydration period (*p* = 0.14). As illustrated in Figure 5, ratings of perceived exertion (A), thirst (B) and heat stress (C) all continuously increased during the TT (all *p* < 0.01), but no condition effect was observed (all *p* > 0.17). Only the pattern of change in the rating of perceived exertion differed between conditions across time (interaction effect, *p* = 0.008), but post-hoc analyses revealed no significant differences. The mean rating of perceived exertion during the TT was not different between conditions (GIH: 15.0 ± 1.3; EUH: 15.5 ± 1.6; *p* = 0.17, effect size = −0.31). Abdominal bloating and pain did not differ between conditions at the end of the hydration period and were representative of baseline levels.

### 3.5. Performance

**Figure 6:**
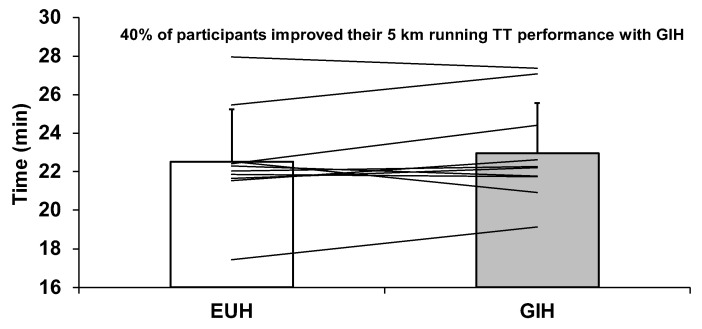
Average and individual performances for the 5 km running time-trial with EUH and GIH. The lines represent changes in individual performances. EUH = euhydrated; GIH = glycerol-induced hyperhydration.

No order effect was detected (*p* = 0.39). There was no relationship between the changes in baseline hydration status and changes in 5-km TT performance (*r* = −0.40, *p* = 0.26). Moreover, in the current study, we observed no relationship (*r* = −0.38, *p* = 0.29) between the 5-km running TT performance achieved with EUH and $\dot{V}$O_2max_. As shown in Figure 6, GIH was associated with a non-significant increase in 5-km TT performance time compared with EUH (GIH: 22.95 ± 2.62; EUH: 22.52 ± 2.74 min, *p* = 0.275, Δ of 2.2 ± 5.1%). The associated effect size was small with a value of 0.16. Only four participants improved their 5-km running TT performance with GIH, with a mean decrease in time of 42.6 ± 37.8 s, compared with EUH. On the other hand, the remaining six participants were 72.2 ± 41.1 s slower with GIH compared with EUH.

## 4. Discussion

The objective of this study was to determine the effect of pre-exercise GIH on fluid balance, physiological and perceptual responses, as well as 5-km running TT performance, under warm conditions in recreationally trained individuals. In this specific population, we expected that the large bolus of fluid ingested with glycerol before exercise would decrease running TT performance time as a result of creating an optimal increase in plasma volume coupled with the anticipated decrease in GI temperature associated with the provision of cold water. Although GIH produced the ideal forecasted theoretical increase in total body water and plasma volume, it did not improve 5 km TT performance nor significantly decrease GI temperature during exercise. Results of the present study fill a void in the literature given that it is the first to examine the impact of GIH on 5-km running TT performance and one of the few to have focused on performance in recreationally active individuals. Indeed, most of the past performance-related GIH studies concentrated on cycling [15,21,33,34,35], triathlon [36] and running performance ≥ 10 km [37,38] in a population of endurance-trained individuals (see the review article by van Rosendal et al. [39]). However, it must be borne in mind that, with the exception of Goulet et al. [15] and, under occupational settings, Latzka et al. [40], the aforementioned studies compared the impact of GIH to that of water-induced hyperhydration, whereas ours compared the effect of GIH to EUH, which is conceptually and fundamentally different. Indeed, to be of any interest for athletes, results of studies looking at hyperhydration on performance must include an EUH condition; for not doing it will not provide any clues as to how this technique may help performance under real-life conditions, where one is interested to understand how hyperhydration compares with EUH. Our results will therefore be helpful for, and provide guidance to, physiologists, coaches, nutritionists and sports physicians in the counseling of recreationally active individuals.

### 4.1. Fluid Retention

In the present study, GIH resulted in an absolute increase in fluid retention of the order of 727 mL. This amount of fluid retention is ~180 mL lower than those observed at minute 120 in previous studies conducted in our laboratory, which used an identical GIH administration protocol [5,6,7]. It is unclear why fluid retention was lower in the current study as, similar to our previous studies, GIH was provided based on FFM levels. One possibility may be related to our sample size, which comprised significantly fewer participants. Having combined glycerol with sodium would have produced a much greater increase in fluid retention and plasma volume expansion than those observed in the current study [5]. However, we quickly eliminated this option as adding sodium to GIH may have resulted in a too severe hemodilution, and consequently, a decrease in arterial O_2_ content [10]. Any fluid ingested before exercise has the potential to induce abdominal pain during exercise if it is not totally integrated within the body at the time of exercise onset [41]. Our aggressive provision of fluid during the first 60 min of the hydration period coupled with the fast integration of glycerol within the body likely allowed GIH to be totally emptied by the stomach and absorbed by the intestine at the time of the initiation of the TT [42,43]. The robust increase in plasma volume along with no observed abdominal pain or bloating at the end of the hydration period supports our assertion. Therefore, from a fluid balance perspective, the use of this GIH protocol prior to a 5-km running TT is suitable.

### 4.2. Plasma Volume Change, Hemoglobin Concentration and Exercise Performance

By the end of the GIH protocol plasma volume had increased by an average of 6.7%. This change in plasma volume is on average 2 points lower than what we observed in our previous studies at the 120 min timepoint [5,6,7]. This observation fits with the concomitant lowest fluid retention we also observed in the current study. Immediately following exercise, plasma volume had decreased to −7.3% with GIH, such that the mean overall change in plasma volume compared to baseline during exercise with this condition was of the order of only −0.4%. This observation fits with the mean hemoglobin concentration observed during exercise with GIH (15.2 g/dL), which is in line with the baseline hemoglobin concentration measured in our subjects (results not shown) in this condition. Thus, from those values, it is reasonable to pretend that, on average, GIH did not significantly modify systemic oxygen delivery to the muscles, contrary to what we had initially anticipated, based on Coyle et al.’s [10] observations. On the other hand, with EUH, the mean decline in plasma volume during exercise compared to baseline was of the order of −7.9%, which was accompanied by a mean increase in heart rate of 4 bpm compared with GIH. Mean hemoglobin concentration during exercise was 15.9 g/dL with EUH. From these numbers, a question arises: Did a hydration condition provide an advantage in terms of systemic oxygen delivery to the muscles during exercise? Our subjects’ mean $\dot{V}$O_2max_ was 3330 mL/min with an associated maximal heart rate of 190 bpm. Assuming a % oxygen extraction of 75%/20 mL oxygen/100 mL of blood at $\dot{V}$O_2max_ [44], then the estimated stroke volume was ~117 mL/b. Let us further assume that during exercise this stroke volume was altered proportionally to the mean changes in plasma volume with GIH and EUH. Then, oxygen delivery to the periphery would have been 4176 mL/min with GIH (176 (mean heart rate during exercise) × 116.5 (mean stroke volume during exercise)/100 × 15.2 (mean hemoglobin concentration during exercise) × 1.34 (assumed mL oxygen/gram of hemoglobin), compared to 4158 mL/min with EUH (180 bpm × 108.4 mL/b/100 × 15.9 g/dL × 1.34). Assuming a % oxygen extraction of 71%/20 mL oxygen/100 mL of blood during the TT [44], then oxygen uptake would have been 2965 mL/min with GIH, compared to 2952 mL/min with EUH. These numbers suggest that GIH and EUH are likely to have a similar effect on oxygen uptake during a 5-km TT.

### 4.3. Gastro-Intestinal Temperature

As expected, the ingestion of a large bolus of cold water with glycerol decreased GI temperature. The peak difference in GI temperature between GIH and EUH occurred between min 60 and 80, reaching ~0.5 °C. However, by the end of the warmup period, the difference between conditions had decreased to 0.2 °C; this gap persisted until the end of the TT. This observation indicates that, by the time the TT started, the cooling advantage that GIH may have provided during exercise had considerably diminished. From a thermoregulatory standpoint, then, it may have been advantageous for participants to initiate the TT at minute 70. However, at this moment, GIH would not have been totally integrated within the body, which may have predisposed participants to develop gastrointestinal problems during the TT [41]. Moreover, by that time, the change in plasma volume had not reached its zenith; therefore, cardiac functions would not have been optimized during the TT. Our findings suggest that the current GIH protocol confers only a modest effect on GI temperature, and the observed magnitude of the change is unlikely to translate into a performance advantage as a variation of 0.2 °C is within the normal daily fluctuation in core body temperature [45]. Future studies need to find strategies to improve the cooling efficiency of this hydration strategy. An appealing avenue may be to combine GIH with the use of a cooling vest during the last 20 min of the protocol as well as during the warmup period. In that respect, Arngrïmsson et al. [46] demonstrated that wearing a cooling vest during a 38-min-long warmup period improved subsequent 5-km running TT performance in the heat.

### 4.4. Exercise Performance

Glycerol-induced hyperhydration did not improve 5-km TT running performance. Several explanations may be provided for this observation. (1) Based on our calculations, the increase in oxygen uptake provided by GIH would have been too small to confer a performance advantage over EUH during the TT. (2) The fluid load ingested with GIH increased BM, which may have reduced running economy, thereby canceling out the slightly improved O_2_ delivery to the muscles [47]. (3) The lack of ergogenic effect of GIH could also possibly be due to the fact that the participants in the current study were training recreationally and had little running experience. Hence, although GIH may have placed them in an ideal physiological state to run a faster 5 km than with EUH, the lack of an experientially developed running-related performance template that would have enabled them to optimize running pace according to the available physiological resources may have prevented any improvement in performance [48]. The fact that thermal comfort and perceived exertion were lower with GIH than EUH through most parts of the TT, coupled with the reduced heart rate and GI temperature observed through all of the TT, supports this idea.

### 4.5. Study Limitations

This study has some limitations worth mentioning. A placebo effect cannot be ruled out. Indeed, personal viewpoints may have led some participants to believe that beginning an exercise while euhydrated may be better than GIH [49]. However, a true placebo, within the context of the current study, cannot blind for hyperhydration as diuresis with GIH is disproportionally more important than with EUH. Results mostly apply to men, as only 1 woman out of our sample size of 10 (10% of the sample) participated in the study. It would have been a better option to measure esophageal temperature instead of gastrointestinal temperature. Indeed, given that the latter responds less rapidly than the former [50] we may have missed a GIH effect on core body temperature. However, GI temperature measurement was preferred because it is less invasive, facilitates recruitment and is associated with no discomfort compared to esophageal temperature measurement. Plasma volume was not directly assessed. Finally, we did not assess the impact of GIH on $\dot{V}$O_2max_.

## 5. Conclusions

In conclusion, this study demonstrates that despite significantly increasing body water and plasma volume, GIH does not improve 5-km running TT performance in the heat in recreationally active individuals. This study is pertinent. In fact, 1) it is the first to examine the impact of GIH on 5-km TT running performance in comparison to a EUH condition and; 2) very few GIH-related studies have focused on performance in recreationally active individuals. There is a genuine interest in studying this population since the vast majority of participants in running events are training on a recreational basis [51].

## Figures and Tables

**Table 1:** Physiological variables and urine and blood markers of the participants at their arrival at the laboratory.

Variables	EUH	GIH	*p*-Value
Body mass (kg)	68.8 ± 8.8	69.1 ± 8.7	0.051
Urine specific gravity (g/mL)	1.008 ± 0.004	1.013 ± 0.005	0.035
Urine osmolality (mOsmol/kg)	391 ± 303	522 ± 282	0.066
Blood osmolality (mOsmol/kg)	296.5 ± 5.9	297.6 ± 16.7	0.781
Blood natremia (mmol/L) *	139.3 ± 2.3	139.7 ± 3.1	0.540

Values are presented as mean ± SD, * *n* = 9.

## Data Availability

The data will be made available from the corresponding author upon reasonable request.
